# Repeated intestinal perforations in vascular Ehlers-Danlos syndrome: a case report of a novel mutation in the *COL3A1* gene

**DOI:** 10.1186/s40792-023-01643-6

**Published:** 2023-05-12

**Authors:** Taichi Horino, Yuji Miyamoto, Mayuko Ohuchi, Katsuhiro Ogawa, Naoya Yoshida, Takatoshi Ishiko, Chieko Kukinaka, Rumi Sasaki, Takashi Ohba, Hideo Baba

**Affiliations:** 1 Department of Gastroenterological Surgery, Graduate School of Medical Sciences, Kumamoto University, 1-1-1 Honjo, Kumamoto, 860-8556 Japan; 2 Department of Gastroenterological Surgery, Kumamoto Kenhoku Hospital, Tamana, Kumamoto Japan; 3 Department of “Development of Nursing Practice”, Graduate School of Medical Sciences, Kumamoto University, Kumamoto, Japan; 4 Department of Obstetrics and Gynecology, Faculty of Life Sciences, Kumamoto University, Kumamoto, Japan

**Keywords:** Ehlers-Danlos syndrome, Autosomal dominant, Intestinal perforation

## Abstract

**Background:**

Ehlers-Danlos syndrome is an inherited connective-tissue disorder characterized by skin hyperextensibility, joint hypermobility, and tissue fragility. Intestinal perforation is one of the fatal manifestations of this syndrome, and its management is complicated.

**Case presentation:**

A 58-year-old woman with a familial history of Ehlers-Danlos syndrome visited the emergency department due to a sudden onset of lower abdominal pain. Plain abdominal computed tomography showed abdominal free air. We found a perforated descending colon and subsequently resected this lesion and performed ileostomy. Fifty-one days after this first operation, the patient had transverse colon perforation and thus underwent the Hartmann procedure as the second operation. In addition, she was diagnosed with small bowel perforation 53 days after the first operation and consequently underwent a third operation—partial resection of the jejunum with functional end-to-end anastomosis. Fifty-eight days after the first operation, she complained of acute abdominal pain. Plain abdominal computed tomography showed fluid collection near the jejunojejunal anastomosis. We detected dehiscence at the entry hole of the linear stapler during the operation and thus performed partial resection of the affected jejunum, followed by jejunostomy. The postoperative course of the fourth operation was uneventful. Genetic testing revealed a novel missense mutation (c.2095G>T, p.Gly699Cys) in the *COL3A1* gene, which is presumed to be a pathogenic variant of vascular Ehlers-Danlos syndrome.

**Conclusion:**

Vascular Ehlers-Danlos syndrome should be considered in the case of repeated intestinal perforation. The identified missense mutation in the *COL3A1* gene (c.2095G>T, p.Gly699Cys) might be a novel pathogenic variation causing vascular Ehlers-Danlos syndrome. Careful postoperative screening and multidisciplinary management are required.

## Introduction

Ehlers-Danlos syndrome (EDS) is an inherited connective-tissue disorder characterized by skin hyperextensibility, joint hypermobility, and tissue fragility [1]. Vascular EDS, also known as type IV EDS, represents 5–10% of patients with EDS. The complications associated with type IV EDS, such as arterial, digestive, and uterine complications, including intestinal perforation, rarely manifest in other types of EDS [2].

Although several operative cases of sigmoid perforation in EDS patients have been reported [3, 4], reports of successfully treated cases requiring multiple operations are rare. Herein, we report the successful treatment of the repeated intestinal perforations in a case of vascular EDS with a novel *COL3A1* mutation via multiple surgical interventions.

## Case presentation

A 58-year-old woman visited the emergency department due to a sudden onset of lower abdominal pain. She had a history of appendectomy, cerebral hemorrhage, and hypertension controlled by an anti-hypertensive drug. Her son had a history of EDS and died from hepatic vessel rupture. However, she had not been examined for EDS. The patient had no characteristic facial features of EDS. Plain abdominal computed tomography (CT) showed abdominal free air with a small amount of effusion surrounding the edematous descending colon. The preoperative diagnosis was panperitonitis due to the perforation of the descending colon, and emergency surgery was performed. We found a perforated descending colon with contaminated ascites and subsequently resected the lesion and performed ileostomy (first operation).

Her initial postoperative course was typical. However, she had perforation in the transverse colon 51 days post-operation and thus underwent the Hartmann procedure (second operation). In addition, she was diagnosed with small-bowel perforation 53 days after the first operation, and thus partial resection of the jejunum with functional end-to-end anastomosis (FEEA) was performed (third operation).

**Fig. 1:**
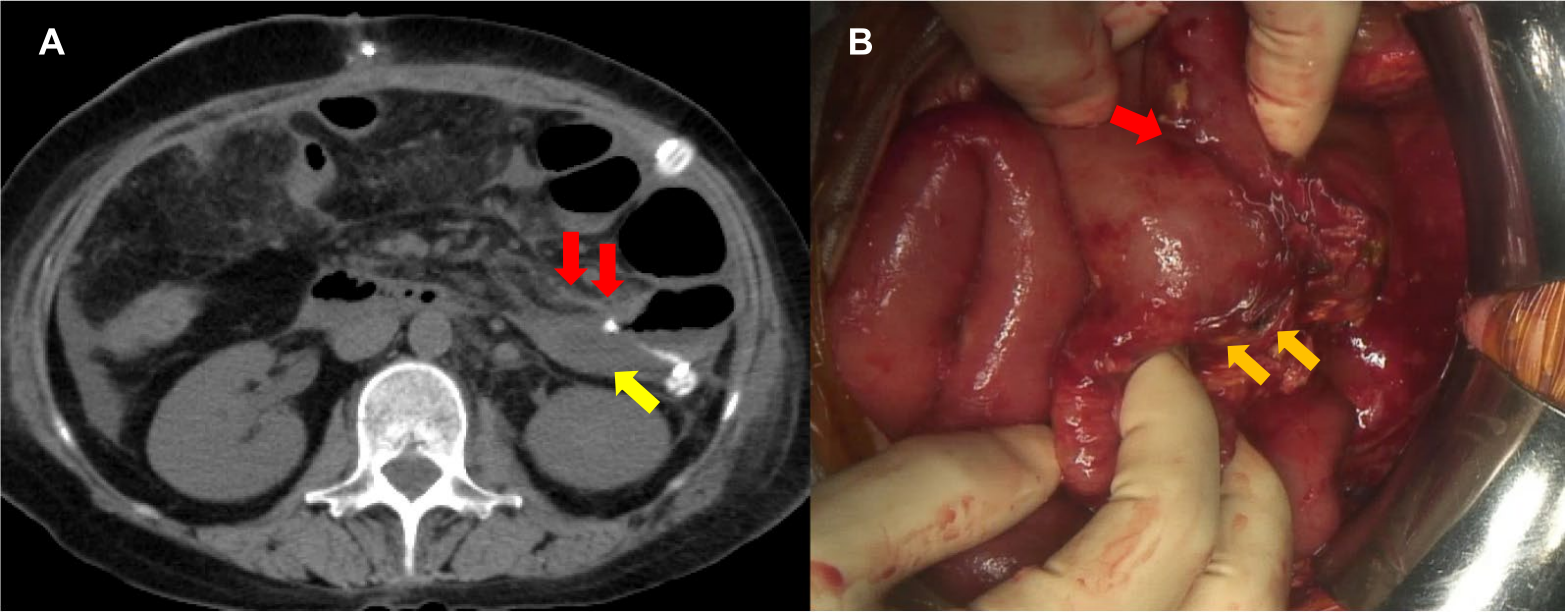
**A** Abdominal computed tomography showing fluid collection (yellow arrow) near the jejunojejunal anastomosis (red arrows). **B** Intraoperative image showing dehiscence at the entry hole of the linear stapler (orange arrows) near the jejunojejunal anastomosis (red arrow)

**Fig. 2:**
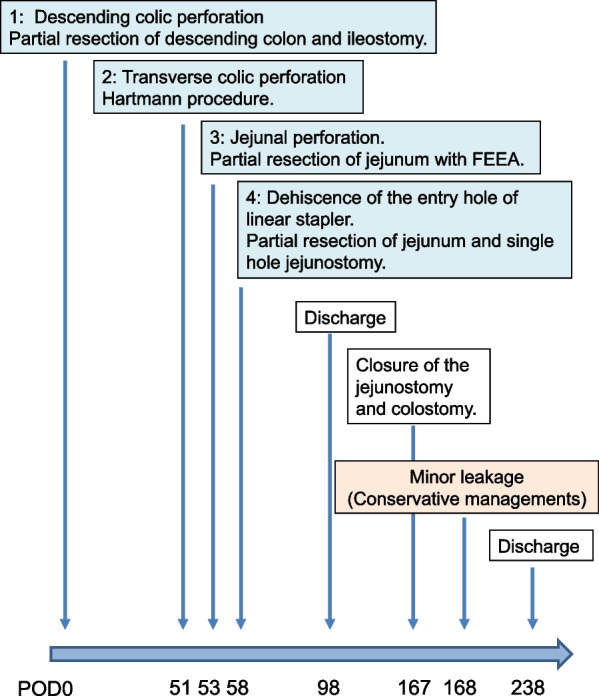
Clinical course of the present case. We performed four operations to treat the repeated intestinal perforations

Fifty-eight days after the first operation, she complained of acute abdominal pain. A bile-contaminated fluid was detected in the drain near the jejunojejunal anastomosis. In addition, plain abdominal CT showed fluid collection near the jejunojejunal anastomosis (Fig. 1A). Therefore, anastomotic leakage was suspected, and we performed another operation (fourth operation). During this operation, we detected dehiscence at the entry hole of the linear stapler used for FEEA (Fig. 1B). However, there were no signs of anastomotic leakage. Therefore, we partially resected the affected jejunum and performed single-hole jejunostomy. The postoperative course of the fourth operation was uneventful, and the patient was discharged from our hospital 98 days after the first operation. Two months later, we performed the closure of the jejunostomy, followed by colostomy. Minor leakage suspected to be caused by anastomotic failure of closure point occurred the next day but was successfully treated via conservative management (Fig. 2).

Genetic examination of a blood sample from the patient revealed a missense mutation in the *COL3A1* gene (c.2095G>T, p.Gly699Cys). Moreover, the same missense mutation was detected in a blood sample from his son, who had suddenly died from hepatic vessel rupture. This mutation is not registered in the Clinvar database, but a similar mutation (c.2095G>C, p.Gly699Arg and c.2095G>A, p.Gly699Asp) has been reported as a pathogenic mutation of EDS and registered in the Clinvar database [5]. Based on the clinical course of the present case, which is strongly associated with EDS, we concluded this novel variant as likely pathogenic. During the follow-up 22 months after the first operation, the patient was found free of intestinal perforation.

**Fig. 3:**
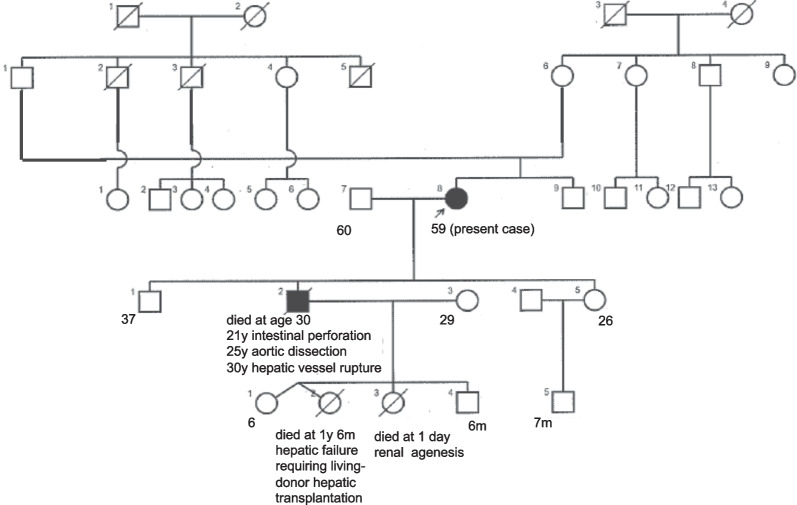
Family tree of the patient. Males and females are represented by squares, and circles, respectively. Mating is shown using a horizontal line between the partners. Offspring symbols are connected in a row beneath the mated pair. The blackened symbol indicates those with the likely pathogenic *COL3A1* variant causing EDS. The small black arrow and diagonal line show the patient and her deceased son, respectively

We also collected detailed information about the family history of the patient. Her family tree is shown in Fig. 3. Among all the immediate family members assessed, only the patient and her son had apparent symptoms of EDS. Two of her grandchildren died from hepatic failure and renal agenesis in their infancy, but there was no clear relationship between their disorders and EDS. We provided the patient and family members with a multidisciplinary support involving genetic counselors during the follow-up period.

## Discussion

Digestive complications, including intestinal perforation, are one of the characteristics of EDS. Most perforation cases occur in the sigmoid colon, but the small intestine is occasionally affected [2]. More than 200 cases of spontaneous intestinal perforation in EDS have been reported in the English language [3]. In addition, similar to our case, there are multiple reports of cases of EDS diagnosed after intestinal perforation [6, 7]. However, reports of successfully treated cases requiring multiple operations are rare. Accordingly, this case is noteworthy, because it required four surgical interventions for the repeated intestinal perforations. Moreover, the missense mutation detected in the present case is presumably a pathogenic variation causing vascular EDS.

In 2017, the international EDS Consortium proposed new classification of EDS which divided it to 13 subtypes [8]. Table 1 shows inheritance pattern and clinical features of each subtypes. Vascular EDS, the fourth subtype, is a rare autosomal dominant disorder, with the estimated prevalence of 1/50,000 to 1/20,000 [9]. The characteristic manifestations of vascular EDS include arterial aneurysm, dissection, and rupture, as well as intestinal perforation and rupture of the gravid uterus. EDS results from pathogenic variants of the *COL3A1* gene, which encodes the chains of type III procollagen, a major protein in the vascular walls and hollow organs.

**Table 1:** Subtypes of EDS

	Subtypes	IP	Major genetic basis	Protein	Major clinical features
1	Classical	AD	*COL5A1*	Type V collagen Type I collagen	Skin hyperextensibility Atrophic scarring Generalized joint hypermobility
2	Classical-like	AR	*TNXB*	Tenascin XB	Skin hyperextensibility Generalized joint hypermobility Easy bruisable skin
3	Cardiac-valvular	AR	*COL1A2*	Type I collagen	Severe valvular diseases Skin involvement Joint hypermobility
4	Vascular	AD	*COL3A1*	Type III collagen Type I collagen	Arterial rupture at a young age Spontaneous colon perforation Uterine rupture during delivery Carotid–cavernous sinus fistula
5	Hypermobile	AD	Unknown	Unknown	Generalized joint hypermobility Mild skin hyperextensibility
6	Arthrochalasia	AD	*COL1A1, COL1A2*	Type I collagen	Congenital bilateral hip dislocation Severe joint hypermobility Skin hyperextensibility
7	Dermatosparaxis	AR	*ADAMTS2*	ADAMTS-2	Extreme skin fragility Characteristic craniofacial features
8	Kyphoscoliotic	AR	*PLOD1, FKBP14*	LH1, FKBP22	Congenital muscle hypotonia Congenital or early onset kyphoscoliosis Generalized joint hypermobility
9	Brittle Cornea syndrome	AR	*ZNF469, PRDM5*	ZNF469, PRDM5	Thin cornea Progressive keratoconus and keratoglobus Blue sclerae
10	Spndylodysplastic	AR	*B4GALT7, B3GALT6,* *SLC39A13*	β4GalT7, β3GalT6, ZIP13	Short stature Muscle hypotonia Bowing of limbs
11	Musculocoontractural	AR	*CHST14, DSE*	D4ST1, DSE	Congenital multiple contractures, Characteristic craniofacial features, Cutaneous features
12	Myopathic	AD or AR	*COL12A1*	Type XII collagen	Congenital muscle hypotonia Proximal joint contractures Hypermobility of distal joints
13	Periodontal	AD	*C1R, C1S*	C1r, C1s	Severe and intractable periodontitis Lack of attached gingiva Pretibial plaques

AD: autosomal dominant; AR: autosomal recessive; IP: inheritance pattern

Following the guidelines of this proposal of the international EDS Consortium [8], we genetically examined the patient and consequently identified that she had a missense mutation in the *COL3A1* gene (c.2095G>T, p.Gly699Cys). Pepin et al. have reported 410 mutations of *COL3A1* gene including c.2095G>C, p.Gly699Arg and c.2095G>A, p.Gly699Asp. Given the association of these two mutations with EDS and their similarity with the mutation we detected in the present case, we concluded that the mutation in the present case is likely a pathogenic variation causing EDS.

Byers et al. have reported that 31% of the vascular EDS cases experience repeated colonic perforation. The same authors have also suggested that protracted wound healing and anastomotic failure might occur due to the vulnerability of the connective tissue and vessels caused by the mutation of the *COL3A1* gene [9]. The present case necessitated four operations. The first, second, and third operations were required due to the idiopathic perforation of the intestine. The last operation was required due to the dehiscence at the entry hole of the linear stapler, reflecting protracted wound healing (Fig. 2).

Given the report of Byers et al., total colectomy can be used to avoid repeated colonic perforation. Moreover, Germain et al. suggested total colectomy with ileostomy and closure of the rectal stump to cope with recurrent intestinal perforations, because perforations in the small intestine are rare [2, 10]. Nevertheless, we experienced jejunal perforation in the present case, which required a second operation. Since the patient was otherwise a healthy woman in her 50s and her preoperative performance status was good, we did not select total colectomy. Therefore, the treatment strategy per patient should be carefully assessed based on their age, physical status, and opinion.

Appropriate screening and prevention methods for intestinal perforations in patients with vascular EDS are still unclear [3]. Pepin et al. have reported that the median life span of patients with vascular EDS is 48 years (range 6–73 years), mainly due to vascular rupture [11]. The same authors have also reported that the prognosis after treatment is still poor. Prolonged postoperative monitoring via non-invasive imaging techniques, including doppler ultrasound, CT angiography with low radiation alternatives, or magnetic resonance angiography, should be performed. In addition, the patient and family members should be provided with appropriate guidance [7].

An international group of specialists have reached into a consensus of recommendations for vascular EDS patients, including lifestyle modifications to minimize injury, maintaining blood pressure within the normal range [9], and carrying a medical attention bracelet and documents noting the information about the condition [12]. This case required multidisciplinary management including physicians, surgeons, and genetic counselors. Because EDS is an autosomal dominant disorder, the supports for not only the patient but also the family members are important. Centralizing management at centers of experience is also essential. In the present case, we informed the patient of the circumstances and encouraged her to contact us if she experienced a sudden pain. We also carefully continued outpatient follow-up for the treatment of her hypertension. During her follow-up periods of 22 months after the initial surgery, she lives without intestinal perforations.

## Conclusions

We experienced a vascular EDS case requiring four operations due to idiopathic intestinal perforation and protracted wound healing. The missense *COL3A1* mutation (c.2095G>T, p.Gly699Cys) found in this case might be a novel pathogenic variation causing vascular EDS. Vascular EDS should be considered in the case of repeated intestinal perforation. In addition, careful screening with multidisciplinary management is required after surgery.

## Data Availability

All data generated or analyzed during this study are included in this published article.

## Abbreviations

CT: Computed tomography
EDS: Ehlers-Danlos syndrome
FEEA: Functional end-to-end anastomosis
